# Anti-CTGF Single-Chain Variable Fragment Dimers Inhibit Human Airway Smooth Muscle (ASM) Cell Proliferation by Down-Regulating p-Akt and p-mTOR Levels

**DOI:** 10.1371/journal.pone.0113980

**Published:** 2014-12-05

**Authors:** Wei Gao, Liting Cai, Xudong Xu, Juxiang Fan, Xiulei Xue, Xuejiao Yan, Qinrong Qu, Xihua Wang, Chen Zhang, Guoqiu Wu

**Affiliations:** 1 Medical School, Southeast University, Nanjing 210009, China; 2 Department of Biological engineering, Southeast University, Nanjing 210009, China; 3 Department of Respiration, Zhongda Hospital, Southeast University, Nanjing 210009, China; 4 Center of Clinical Laboratory Medicine, Zhongda Hospital, Southeast University, Nanjing 210009, China; Helmholtz Zentrum München/Ludwig-Maximilians-University Munich, Germany

## Abstract

Connective tissue growth factor (CTGF) contributes to airway smooth muscle (ASM) cell hyperplasia in asthma. Humanized single-chain variable fragment antibody (scFv) was well characterized as a CTGF antagonist in the differentiation of fibroblast into myofibroblast and pulmonary fibrosis in our previous studies. To further improve the bioactivity of scFv, we constructed a plasmid to express scFv-linker-matrilin-6×His fusion proteins that could self-assemble into the scFv dimers by disulfide bonds in matrilin under non-reducing conditions. An immunoreactivity assay demonstrated that the scFv dimer could highly bind to CTGF in a concentration-dependent manner. The MTT and EdU assay results revealed that CTGF (≥10 ng/mL) promoted the proliferation of ASM cells, and this effect was inhibited when the cells were treated with anti-CTGF scFv dimer. The western blot analysis results showed that increased phosphorylation of Akt and mTOR induced by CTGF could be suppressed by this scFv dimer. Based on these findings, anti-CTGF scFv dimer may be a potential agent for the prevention of airway remodeling in asthma.

## Introduction

Asthma is considered an inflammatory disease of the airway that responds poorly to current therapies and affects more than 300 million individuals worldwide [1]. Severe persistent asthma can result in structural changes in the airway, such as thickening of the basement membrane, airway smooth muscle (ASM) hyperplasia and/or hypertrophy, changes in the extracellular matrix (ECM) composition and an increase in the number of blood vessels, which are collectively referred to as airway remodeling [2]–[5].

Transforming growth factor-β (TGF-β) is commonly associated with various disorders involving inflammation and repair, such as asthma and chronic obstructive airway disease. Previous studies have shown that TGF-β expression is up-regulated in bronchial biopsies from patients with asthma and stimulates human ASM cell growth [6], [7]. Connective tissue growth factor (CTGF), a downstream mediator of TGF-β, plays various roles in promoting broad cellular responses, such as proliferation, chemotaxis, adhesion, migration and ECM production, as well as in regulating diverse processes in vivo, such as angiogenesis, differentiation and wound healing [8], [9]. The role of CTGF in ASM cell proliferation and airway remodeling, however, remains unclear [6], [10].

In our previous study, the humanized single-chain variable fragment (scFv) antibody against the CTGF C-terminal domain was obtained from a phage display human antibody library [11], and it was shown that it may play a potential role in attenuating pulmonary fibrosis in mice [12]. It has been shown that the recombinant anti-CTGF scFv antibody can neutralize the biological activity of CTGF and decrease the differentiation of fibroblast into myofibroblast by inhibiting Akt phosphorylation [13].

Matrilin-1, previously referred to as cartilage matrix protein (CMP), is composed of three functional domains and a C-terminal module that consists of 42 amino acids, which forms a coiled-coil structure that allows the subunits to assemble [14]. Matrilin-1 may play a role in stabilizing the extracellular matrix structure because it has been shown that it can self-associate into supramolecular structures, which results in the formation of filamentous networks [14]–[16]. If the matrilin-1 module is incorporated into the scFv antibody C-terminal and a scFv antibody dimer forms through the covalent linking of two scFv monomers, the biological activity of the anti-CTGF scFv antibody may be enhanced. In this study, we designed a plasmid named pET28a-scFv-matrilin and an anti-CTGF scFv antibody dimer was expressed and purified. Next, we investigated the effects of this dimer on the ASM cell proliferation and the expression of phosphorylated Akt (protein kinase B) (p-AKt) and phosphorylated mTOR (mammalian target of rapamycin) (p-mTOR) induced by CTGF in human ASM cells.

## Materials and Methods

### Materials and reagents

Human ASM cells were purchased from ScienCell (Corte Del Cedro Carlsbad, CA, USA). *E. coli* BL21(DE3) and the pET28(a)+ vector were purchased from Novagen (Germany). The recombinant human CTGF/CCN2 C-terminal domain, Cyr61/CCN1 and NOV/CCN3 were purchased from PROSPEC (USA). The monoclonal mouse anti-human CTGF/CCN2 C-terminus, Cyr61/CCN1 and NOV/CCN3 monoclonal antibody were purchased from R&D system (USA). The anti-6xHis monoclonal antibody was from abcam (UK). The Cell_Light EdU DNA Cell Proliferation Kit was from Ribobio (China). LY294002 (PI3K inhibitor), rabbit antibodies against Akt, mTOR, β-actin, phosphorylated-Akt (Ser473), and phosphorylated-mTOR, and HRP-goat anti-rabbit IgG were purchased from Cell Signaling (USA). NcoI, XhoI, T4 DNA ligase and Easy Taq DNA polymerase were purchased from TaKaRa (Japan). The BCA protein assay kit was provided by Tiangen (China). Cell culture media were purchased from Gibco BRL (USA). The newborn calf serum was obtained from Shiqing (China). Isopropyl-1-thio-β-D-galactoside (IPTG) was purchased from Sigma-Aldrich. The His-bind resin column and total protein extraction kit were purchased from KeyGEN (China).

### Vector construction

To construct a pET28a-scFv-matrilin plasmid, the nucleotide sequence encoding C-terminal domain of matrilin-1 was inserted into 3′-terminal of scFv antibody sequence, and two sequences was linked by nucleotide sequence encoding GGGSGGGSGS. Based on the amino acid sequence in GenBank, access no. GQ390339 (anti-CTGF scFv), and a previous report (C-terminal domain of matrilin-1) [14], the nucleotide sequence encoding scFv-GGGSGGGSGS-Matrilin was chemically synthesized by Genscript Biotech (Nanjing, China). The resulting product was used as the template in a PCR to incorporate the NcoI and XhoI restriction sites at the 5′ and 3′ ends of the sequence. The amplified product and pET28(a)+ vector were digested with the NcoI and XhoI enzymes for 2 h, gel purified and ligated using T4 DNA ligase to obtain the pET28a-scFv-matrilin plasmid. Finally, the plasmid was verified by DNA sequencing (BioAsia Biology, Shanghai, China).

### Expression and purification of fusion protein

The protein was expressed as previously described [11]. Briefly, a single colony of *E. coli* BL21 containing the pET28a-scFv-matrilin plasmid was inoculated in 5 mL LB medium supplemented with kanamycin (50 µg/mL) at 37°C. When the optical density (OD600) reached 0.6–0.7, the cells were collected and resuspended in 500 mL LB medium containing kanamycin. The temperature was initially maintained at 37°C until the culture OD600 reached 0.6–0.7, and then, the temperature was adjusted to 17°C, IPTG was added to a final concentration of 0.3 mM and the cells were incubated overnight (12 h) for protein expression. After this incubation with shaking, the harvested cells were resuspended in 20 mL of bacterial lysis buffer (50 mM Tris-HCl, 100 mM NaCl, 1 mM EDTA, pH 8.0) and lysed using sonication at 300 W for 50 cycles (5 s on, 5 s off) in an ice-water bath. The supernatant was collected after centrifugation at 10,000×g for 10 min and subjected to a Nickel-affinity chromatography. The purified protein was subjected to 12% sodium dodecyl sulfate-polyacrylamide gel electrophoresis (SDS-PAGE) and 12% native-PAGE, and the results were analyzed using Quantity One software (Bio-Rad, USA). In addition, Western bolt was performed using an anti-6xHis monoclonal antibody as described below.

### Enzyme-linked immuno sorbent assay (ELISA)

The immunoreactivity of the scFv dimer against human CTGF was evaluated using ELISA according to a previously described method [13]. Briefly, 96-well flat-bottom Costar microplates were coated with purified scFv dimer or scFv monomer (100 µL per well, the scFv monomer was produced earlier by our laboratory [13]) at concentrations ranging from 0.0312 to 2 µg/mL and 0.05 M sodium bicarbonate (pH 9.6) and were incubated overnight at 4°C. Next, the wells were blocked in 0.1 M phosphate-buffered saline (PBS, pH 7.4) plus 2% bovine serum albumin and 0.1% Tween-20. After washing with PBS, the wells were incubated with 100 µL of CTGF, Cyr61 or NOV (1 µg/mL) for 2 h at 37°C. Then, 100 µL of anti-CTGF, anti-Cyr61 or anti-NOV monoclonal antibody (1 µg/mL) was added respectively, and the wells incubated for 1 h. After washing, the secondary antibody (HRP-conjugated goat anti-rabbit IgG) in blocking buffer (1∶1,000) was added, and the wells incubated for 1 h. Finally, the tetramethylbenzidine substrate (100 µL/well) was added, and the wells incubated for 10 min. The plates were analyzed using an automated ELISA reader at 450 nm (Bio-Rad, USA).

### Cell culture and MTT assay

Human ASM cells were cultured in six-well plates with Dulbecco's modified Eagle medium (DMEM), supplemented with L-glutamine, 10% newborn calf serum, 100 IU/mL penicillin, and 100 µg/mL streptomycin in a 5% CO_2_ atmosphere at 37°C for 24 h as previously described [17], [18]. Next, the cells were trypsinized and diluted to a concentration of 1×10^3^ cells/100 µL in serum free medium. A total of 100 µL human ASM cells were added to each well of 96-well plates. After incubating overnight, the cells were treated with recombinant human CTGF at a final concentration of 0–30 ng/mL and cultured for 1–5 days. For the proliferation inhibition assay, the cells were incubated in 10 ng/mL CTGF and 100 ng/mL anti-CTGF scFv monomer, 100 ng/mL anti-CTGF scFv dimer, or 1 µg/mL LY294002 for 1–5 days. A total of 20 µL MTT reagent [3-(4,5-Dimethylthiazol-2-yl)-2,5-diphenyltetrazolium bromide, 5 mg/mL] was added to each well, and the cells were incubated at 37°C for 4 h. The medium in each well was then replaced with 150 µL dimethylsulfoxide (DMSO). The plates were incubated on a shaker for 15 min at room temperature, and then the absorbance was measured using an automated ELISA reader (Bio-Rad, USA) at 490 nm.

### 5-ethynyl-2′-deoxyuridine (EdU) assay

DNA synthesis was examined by way of EdU assay. ASM cells were resuspended in fresh complete medium, counted and plated on 0.01% poly-L-lysine-coated 96-well plates at a density of 5×10^4^ cells/mL. After treatment with CTGF in the presence or absence of inhibitors 50 µM EdU was added to allow culture for additional 2 h. ASM cells were fixed with 4% formaldehyde in PBS for 30 min, followed by assay using the EdU kit according to the manufacturer's protocol. The result was expressed as a percentage of maximum absorbance based on three replicates in three independent experiments.

### Western blot

To determine whether CTGF promotes human ASM cell growth through the PI3K signaling pathway, the expression levels of p-Akt/Akt and p-mTOR/mTOR were determined using Western blot analysis. Briefly, ASM cells were cultured in DMEM and exposed to starvation medium for 24 hours before treatment. The cells were divided into the following five treatment groups: the control (saline water) group, CTGF (10 ng/mL) group, CTGF (10 ng/mL)+scFv dimer (100 ng/mL) group, CTGF (10 ng/mL)+LY294002 (1 µg/mL) group, and CTGF (10 ng/mL)+mAb (100 ng/mL) group. For the groups with pharmacological inhibitors, the cells were cultured for 1 h with inhibitor before CTGF was added, and then the cells were cultured for 3 days in the presence of both the inhibitor and CTGF. Cells were lysed using a total protein extraction kit. After centrifugation at 13,000×g for 15 min at 4°C, the supernatant was collected.

Besides, the purified recombinant protein was obtained in the expression and purification of fusion protein section as above and the protein levels were determined using a BCA protein assay kit. Additionally, a 50 µg sample of supernatant was subjected to 10% SDS-PAGE in a Mini-Protean II Electrophoresis Cell (Bio-Rad, USA). The separated proteins were transferred onto a polyvinylidene fluoride (PVDF) membrane (Millipore) in a Mini Trans-Blot chamber set at 200 mA for 1 h with transfer buffer (25 mM Tris-HCl, 192 mM glycine, and 20% methanol). The PVDF membrane was blocked for 1 h in a 5% instant skim milk Tris-buffered saline solution and then incubated in rabbit anti-6×His tag antibody, Akt, mTOR, phosphorylated Akt or phosphorylated mTOR antibody (1∶1,000) overnight at 4°C, and then horseradish peroxidase-linked goat anti-rabbit immunoglobulin G (1∶5,000) was added. Finally, the membrane was washed, the results were visualized using BU-Western Blot Detection System (Biouniquer, China), and the image was obtained using X-ray film exposure (Kodak, Japan). Anti-β-actin monoclonal antibody was used to normalize the protein expression levels. The Quantity One analysis software was used to quantify each band. All the experiments were performed in triplicate.

### Reverse transcription-polymerase chain reaction (RT-PCR)

RT-PCR was used to determine the intracellular level of mTOR mRNA. Total RNA was extracted from cells treating for three days described as above using the TRizol total RNA kit according to the manufacturer's instructions. The total RNA concentration was determined by measuring the absorbance at 260 nm in a photometer (Eppendorf Biophotometer). The ratios of absorption (260/280 nm) for all of the preparations were between 1.8 and 2.0. Aliquots of the RNA samples were subjected to electrophoresis through a 1.0% agarose formaldehyde gel to verify their integrity.

The reverse transcription (RT) reaction mixture for first strand cDNA synthesis consisted of 5 µL 5×RT buffer, 1.25 µL 10 mM dNTPs, 1 µL 200 µM-MLV reverse transcriptase, 5 µL 2.5 µM random hexamer primers, and 2 µg of total RNA in a final volume of 25 µL. RNA samples were denatured at 70°C for 5 min and placed on ice for 5 min with random hexamer primers and dNTP before reverse transcription. The samples were incubated for 1 h at 42°C and 10 min at 95°C, and they were then chilled to 4°C.

For polymerase chain reaction analysis, 2 µL of the RT reaction mixture was used in a 25 µL total reaction volume containing 1.5 U Easy Taq DNA polymerase, 0.25 mM of each dNTP, 2.0 mM MgCl_2_, 1.0 µM each of the forward and reverse primers (5′-CTGGGACTCAAATGTGTGCAGTTC-3′ and 5′-GAACAATAGGGTGAATGATCCGGG-3′) and 10 µL SYBRGreen I dye. The primers used for β-actin were 5′-CACTCTTCCAGCCTTCCTTCC-3′ and 5′-TGCCAGGGTACATGGTGGT-3′. Amplification was performed using 35 cycles of 95°C for 30 s, 50.5°C for 30 s, and 72°C for 45 s in a CFX-96 real time system (BIO-RAD, USA). The cycle threshold (CT) values were measured and calculated using computer software. The relative transcript levels were calculated as x = 2^−ΔΔCT^, in which ΔΔCT = ΔCT_(experiment group)_ – ΔCT_(control group)_ and ΔCT = CT_(mTOR)_- CT_(β-actin)_.

### Statistical analysis

Data are expressed as the means ± SD, and the statistical analysis was conducted using an analysis of variance (ANOVA) followed by a Bonferroni post hoc test. All of the data were analyzed using the SPSS 18.0 statistical software package, and probability values less than 0.05 were considered statistically significant.

## Results

### Construction of the pET28a-scFv-matrilin vector

**Figure 1:**
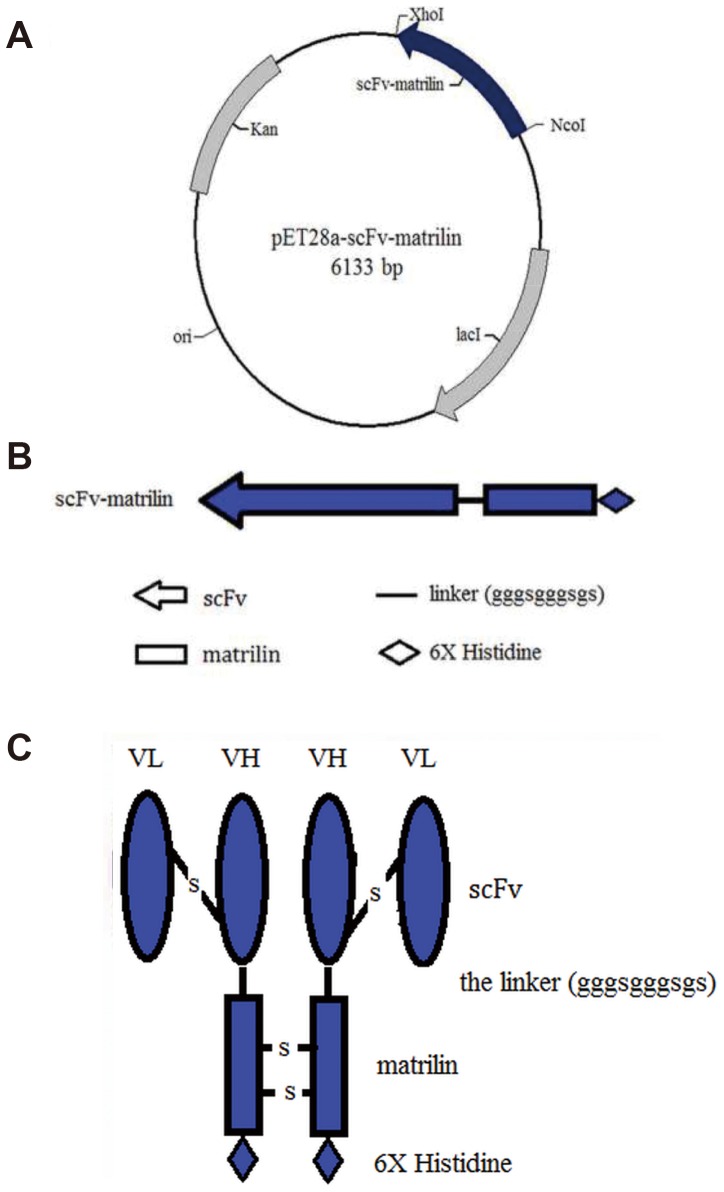
Schematic representations of the expression vector pET28a-scFv-matrilin (A), the scFv-matrilin fusion protein sequence (B) and the scFv dimer structure (C) are shown.

The pET28a-scFv-matrilin plasmid was successfully constructed by ligating the gene encoding 296-amino acid residues with the pET-28a(+) plasmid (Figure 1A), and. the final sequence was scFv-linker-matrilin-6×His (Figure 1B). The sequencing result for the recombinant plasmid was consistent with the predicted sequence. Schematic representation of the scFv dimer structure was shown in Figure 1C.

### Expression and purification of scFv dimer

**Figure 2:**
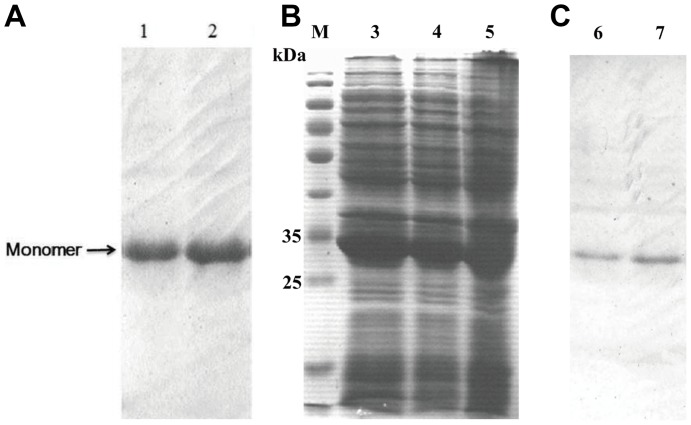
Expression and purification of scFv-matrilin were evaluated using SDS-PAGE and Western blot. M marker, lanes 1, 2 show the fusion protein purified by nickel affinity chromatography, lanes 3, 4 show the supernatant of the cell lysate after IPTG (0.3 mM) induction at 37°C for 12 h, lane 5 shows the supernatant of the cell lysate before IPTG induction, lanes 6, 7 show immunodotting bands with.anti-6xHis tag antibody (C).

**Figure 3:**
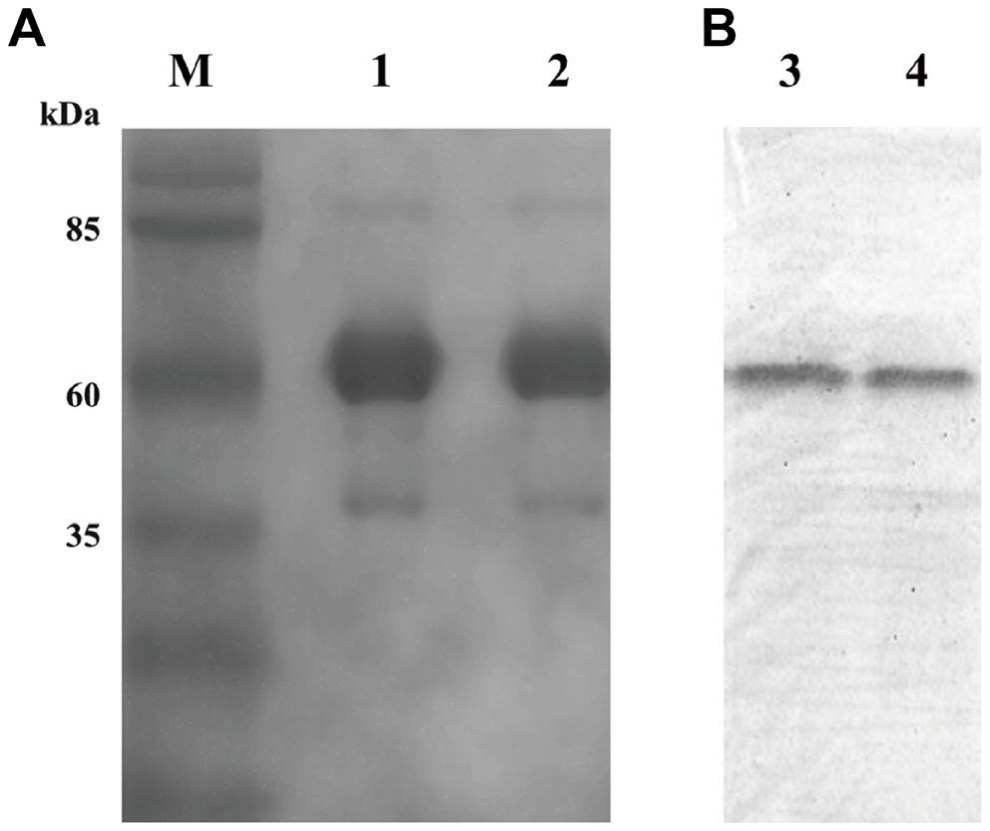
ScFv-matrilin analyzed using native-PAGE and Western blot. M marker, lanes 1, 2 show the fusion protein purified by nickel affinity chromatography, lanes 3, 4 show immunodotting bands with.anti-6xHis tag antibody.

A soluble scFv fusion protein was effectively expressed under optimal condition. The amount of the recombinant protein was more than 40% of the total protein in the supernatant of the cell lysate (Figure 2B, lane 3 and lane 4). After affinity chromatography, the purity of scFv-matrilin was over 95% according to the SDS-PAGE analysis results (Figure 2A, lane 1 and lane 2). The native-PAGE results revealed a main band that was approximate 62 kDa (Figure 3A), which is consistent with calculated size of the scFv-matrilin dimer. Western blot result demonstrated that the recombinant protein specifically combined with the anti- 6xHis monoclonal anbody (Figure 2C, lane 6 and lane 7; Figure 3B).

### Immunoreactivity assay using ELISA

**Figure 4:**
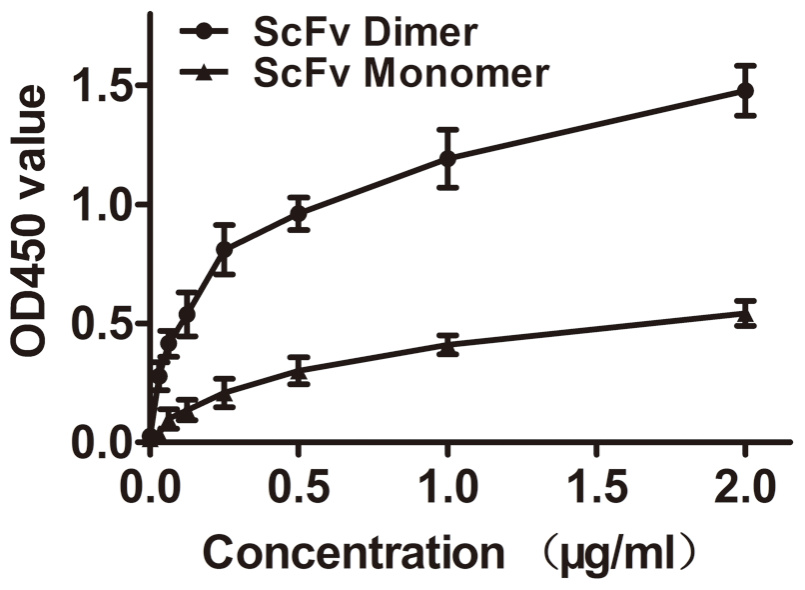
Immunoreactivity assay determined by ELISA. Both the scFv dimer and scFv monomer specifically reacted with CTGF. The regression equations are Y = 0.3633+1.1273X-0.3351X^2^ (R^2^ = 0.938), and Y = 0.0453+0.7185X−0.1960X^2^ (R^2^ = 0.994) for the scFv dimer and scFv monomer, respectively. The OD450 value for the scFv dimer was two times higher than the value for scFv monomer when the concentrations were equal to the CTGF concentration.

The Immunoreactivity of the scFv dimer to CTGF was determined using ELISA. As shown in Figure 4, there was a dose-dependent relationship between the OD450 value and the scFv dimer concentration (Y = 0.3633+1.1273X-0.3351X^2^, R^2^ = 0.938). Concentration for 50% of maximal effect (EC50) was 1.592±0.12 µg/mL. Although a dose-dependent relationship between the OD450 value and scFv concentration was observed (Y = 0.0453+0.7185X−0.1960X^2^, R^2^ = 0.994), the EC50 (3.250±0.83 µg/mL) was two times higher than the EC50 for the scFv dimer when the scFv dimer concentration was equal to the CTGF concentration. Additionally, no cross-reaction was found between the scFv dimer and Cyr61 or NOV, the optical values were less than 0.05 (data not shown).

### Effect of anti-CTGF scFv dimer on ASM cell proliferation

**Figure 5:**
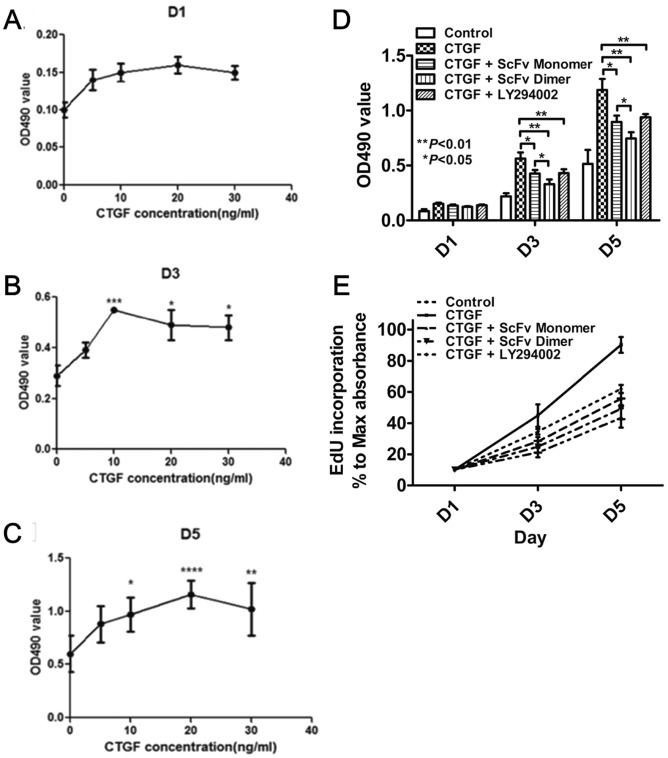
Human ASM cell proliferation after stimulated by CTGF. A. Results from the MTT assay after ASM cells were incubated in CTGF (0–30 ng/mL) for 1 day. B. Results from the MTT assay after ASM cells were incubated in CTGF (0–30 ng/mL) for 3 days. C. Results from the MTT assay after ASM cells were incubated in CTGF (0–30 ng/mL) for 5 days. D. The results from the MTT assay after ASM cells were incubated for 1–5 days in CTGF (10 ng/mL) with or without the scFv monomer, scFv dimer, or LY294002. E. The results from EdU incorporation assay after ASM cells were incubated for 1–5 days in CTGF (10 ng/mL) with or without the scFv monomer, scFv dimer, or LY294002. * p<0.05, ** p<0.01, *** p<0.005, **** p<0.001.

To investigate the effect of CTGF on ASM cell proliferation, the cells were exposed to various concentrations of CTGF (0, 10, 20, 30 and 40 ng/mL) for 1–5 days, and the amount of cell growth was determined using a MTT assay. There was no statistically significant difference in growth among the various concentration groups when the ASM cells were incubated in CTGF for 1 day (p>0.05, Figure 5A). There was, however, a significant increase in cell growth in the CTGF group (≥10 ng/mL) compared with the control group when the cells were cultured for 3 or 5 days (p<0.05, Figure 5B and C). The cell proliferation induced by 10 ng/mL CTGF after a 3 or 5-day incubation period was significantly inhibited in the presence of the scFv monomer, scFv dimer, or LY294002 (Figure 5D). Similar results were observed in EdU incorporation assay (Figure 5E).

### Effect of anti-CTGF scFv dimer on p-Akt and p-mTOR expression

**Figure 6:**
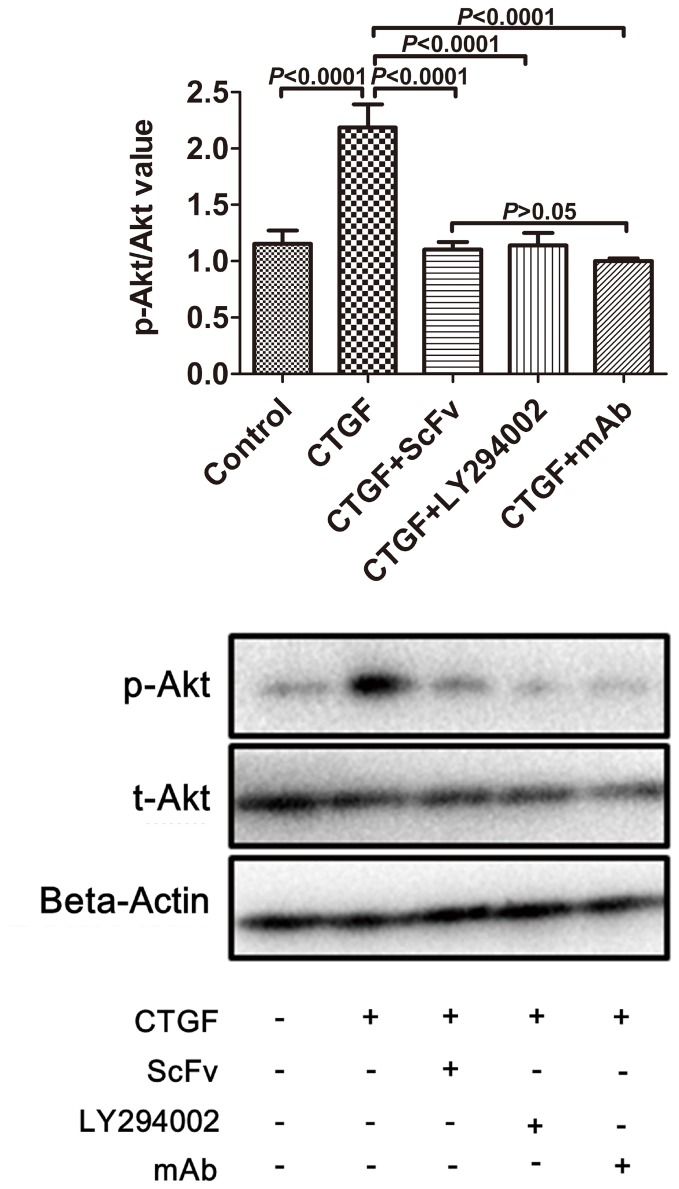
Expression levels of p-Akt/Akt in human ASM cells determined by Western blot analysis. The ratio was higher in cells co-cultured in CTGF (p<0.0001). The increase in the p-Akt level induced by CTGF was significantly inhibited when the cells were treated with scFv dimer, LY294002, or mAb (p<0.001). There was no significant difference between the scFv group and mAb group (p>0.05).

**Figure 7:**
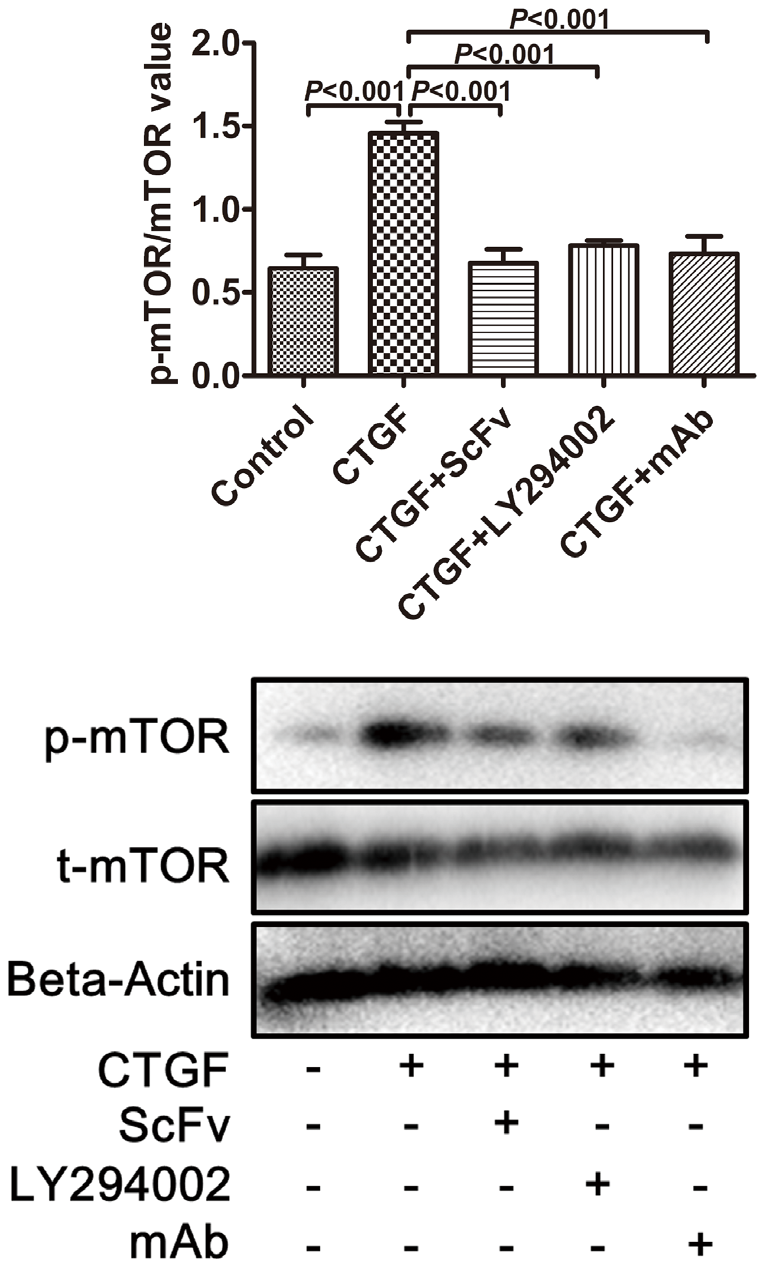
Expression levels of p-mTOR/mTOR in human ASM cells determined by Western blot analysis. The ratio was higher in cells co-cultured in CTGF (p<0.0001). The increase in the p-mTOR induced by CTGF was significantly inhibited when the cells were treated with scFv dimer, LY294002, or mAb (p<0.001). There was no significant difference between the scFv group and mAb group (p>0.05).

To investigate the potential mechanism involved in the anti-CTGF scFv dimer inhibition of ASM cell proliferation, Western blot analysis was used to detect the expression levels of Akt, p-Akt, mTOR, and p-mTOR. As shown in Figure 6 and 7, the p-Akt/Akt and p-mTOR/mTOR ratios significantly increased in the CTGF group compared with the control group (p<0.0001). Additionally, there were statistically significant decreases in the p-Akt/Akt and p-mTOR/mTOR ratios in the CTGF+scFv dimer, CTGF+anti-CTGF monoclonal antibody (mAb) and CTGF+LY294002 groups compared with the CTGF group (p<0.001). There was no significant difference between the CTGF+scFv dimer group and the CTGF+mAb group (p>0.05).

### Intracellular level of mTOR mRNA

**Figure 8:**
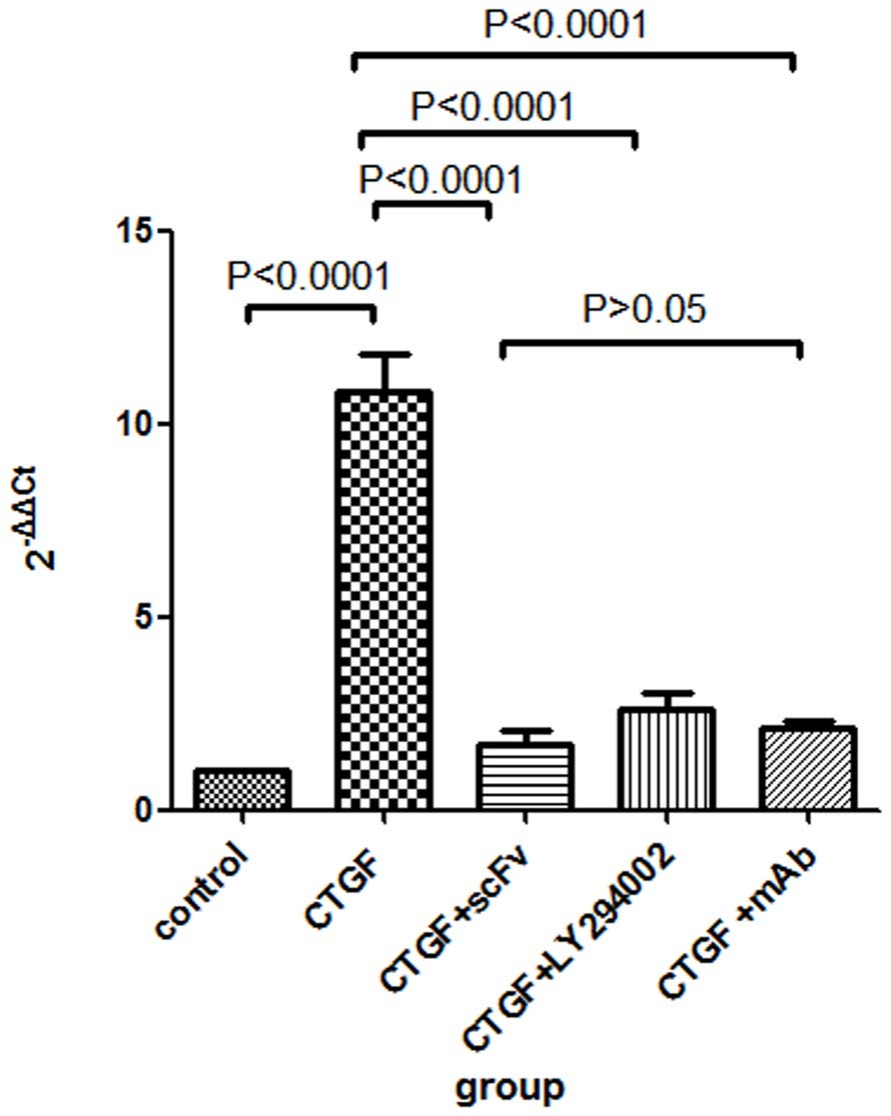
MTOR mRNA level in human ASM cells determined by RT-PCR. All 2^−ΔΔCT^ values were calculated, and the value was assumed to be 1 for the control group. The increase in the mTOR mRNA level in ASM cells was significantly inhibited when the cells were treated with scFv dimer, LY294002, or mAb (p<0.0001). There was no statistically significant difference between the scFv group and mAb group (p>0.05).

Real time RT-PCR was used to determine the mTOR mRNA level in ASM cells. As shown in Figure 8, the 2^−ΔΔCT^ value was significantly higher in the CTGF group compared with the control group (p<0.0001) and significantly lower in the CTGF+scFv dimer, CTGF+scFv mAb and CTGF+LY294002 groups compared with the CTGF group (p<0.0001). No significant difference was observed among the CTGF+scFv dimer group, CTGF+scFv mAb group and CTGF+LY294002 group (p>0.05). These results suggest that the scFv dimer, mAb and LY294002 inhibit the up-regulation in mTOR mRNA expression induced by CTGF in human ASM cells.

## Discussion

CTGF/CCN2, a member of the CCN family, is a small cysteine-rich secreted multi-modular protein that regulates various physiological and pathological processes [19], [20]. It contains four domains: an insulin-like growth factorbinding domain (module I), a Von Willebrand factor type C repeat domain (module II), a thrombospondin type1 repeat domain (module III), and a C-terminal cysteine knot (Cys-knot) (module IV). The N-terminal domain (modules I and II) has been shown to mediate differentiation and collagen synthesis, and the C-terminal domain (modules III and IV) has been shown to regulate cell proliferation [21]–[23]. Recently, it has been shown that ASM cells produce and release CTGF [6], [24], which plays a role in increasing angiogenesis and the accumulation of ECM in the asthmatic airway [25]. The increase in the amount of CTGF released from asthmatic ASM cells may have an autocrine feedback effect that increases the amount of ECM protein released from ASM cells, which would further stimulate the remodeling occurring in the asthmatic airway [25].

ScFv, which contains a complete antigen-binding site (a variable heavy and a variable light domain), is considered a promising antibody in specific medical applications and has certain advantageous qualities: it is a smaller molecule and has less immunogenicity compared with full-length monoclonal antibodies [26]. It has been shown that anti-CTGF scFv significantly attenuates bleomycin (BL)-induced pulmonary fibrosis in mice by inhibiting the CTGF bioactivity [12]. To further improve the avidity of the scFv antibody, we constructed the expression plasmid pET28a-scFv-matrilin, which includes the matrilin-1 C-terminal domain that allows for scFv dimer formation because of the two disulfide bonds that form in the coiled-coil domains (Figure 1). A soluble recombinant scFv dimer with higher purity was obtained by transformation, induction, expression and purification as expected (Figure 2 and 3). The scFv dimer exhibited an excellent immunoreactivity to CTGF by ELISA assay, and the EC50 value was two times lower that of the scFv monomer (Figure 4).

In this study, we found that ≥10 ng/mL CTGF increased ASM cell proliferation, and this effect lasted at least 3–5 days (Figure 5B and C). Furthermore, this proliferation was also inhibited when the cells were treated with anti-CTGF scFv dimer or mAb. It has been shown that TGF-β induces hyperplasia and hypertrophy in ASM cells, and the protein and mRNA expression levels of CTGF are significantly higher in response to TGF-β in asthmatic ASM cells [6], [27]. The combination with these results and our previous result, which demonstrate that the anti-CTGF scFv antibody suppresses the TGF-β-induced differentiation of fibroblast into myofibroblast by inhibiting AKT phosphorylation [13], suggest that TGF-β contributes to the hyperplasia and hypertrophy of ASM cells through a mechanism in which CTGF acts as a downstream factor of TGF-β.

Recent studies investigating the mechanism involved in TGF-β signaling dependent ASM proliferation have demonstrated the importance of several pathways, including the mitogen-activated protein kinase (MAPK) and phosphatidylinositol 3-kinase (PI3K) pathways [28], [29]. PI3K/Akt is a classic signal pathway, and its activation induces the G1/S cell cycle transition [30], [31]. MTOR is a highly conserved serine/threonine kinase that belongs to the PI3K family and is one of the downstream targets of Akt in regulating cell proliferation and protein translation. The evidence suggests that the mTOR pathway is vital for airway and vascular remodeling, which is a key characteristic of severe asthma [32]. We firstly find that CTGF can enhance ASM proliferation in this study, however, the potential mechanism remains to be investigated. Our study shows that the p-Akt/Akt and p-mTOR/mTOR ratios were dramatically higher after ASM cells were cultured in CTGF for 3 days and were significantly lower when the cells were also treated with anti-scFv dimer, anti-CTGF mAb and the specific PI3K inhibitor LY294002 (Figure 6 and 7). The result was similar to that of real-time RT-PCR for mTOR mRNA (Figure 8). The inhibiting effect of the scFv dimer was no different from those of inhibitors LY294002 and mAb (p>0.05). This finding proves the effectiveness of the scFV dimer as a new CTGF antagonist.

Because of culturing cells for three days, we can not distinguish between direct effects and indirect effects, but it seems to be an indirect effect from the data analysis and experimental design. In addition, we found that the increased mTOR mRNA was not parallel to the western blot data in total protein. In fact, the increased total mRNA does not always result in protein high-expression. The mRNA translation is affected by various regulatory factors or inference, such as transcription factors, miRNAs, and external chemical or biological agents [33], [34]. Moreover, many signal molecules such as AKT, ERK, and mTOR are short-life proteins, not structural protein [35], and these signal proteins may be accelerately degraded by the ubiquitin-proteasome system under the regulation or influence of certain factors [36], [37].

Altogether, our results clearly indicate that CTGF may participate in ASM cell proliferation through the PI3K/Akt/mTOR pathway and that the anti-CTGF scFv dimer may inhibit the increased p-Akt and p-mTOR levels induced by CTGF in ASM cells. CTGF is potentially a key factor in developing and maintaining the structural changes that are associated with severe persistent asthma. Based on these findings, it is possible that neutralizing the CTGF bioactivity using a monoclonal antibody, such as the anti-CTGF scFv dimer, may be an effective treatment for minimizing the amount of airway remodeling in asthma patients.
